# Retinal microvascular and structural changes in intracranial hypertension patients correlate with intracranial pressure

**DOI:** 10.1111/cns.14298

**Published:** 2023-06-08

**Authors:** William Robert Kwapong, Le Cao, Ruosu Pan, Hang Wang, Chen Ye, Wendan Tao, Junfeng Liu, Bo Wu

**Affiliations:** ^1^ Department of Neurology, West China Hospital Sichuan University Chengdu China

**Keywords:** intracranial hypertension, intracranial pressure, papilledema, retinal microvasculature, retinal structure

## Abstract

**Aims:**

We aimed to evaluate the retinal microvascular and structural changes in intracranial hypertension (IH) patients compared with an age‐ and sex‐matched control group. We also investigated the association between clinical parameters and retinal changes in IH patients.

**Methods:**

Intracranial hypertension patients were divided into eyes with papilledema (IH‐P) and eyes without papilledema (IH‐WP). IH patients underwent lumbar puncture to measure intracranial pressure (ICP); visual acuity was performed using the Snellen chart. Optical coherence tomography (OCT) was used to image and measure the retinal nerve fiber layer (RNFL) and ganglion cell‐inner plexiform layer (GCIPL) while OCT angiography was used to image and measure the superficial vascular complex (SVC) and deep vascular complex (DVC).

**Results:**

Intracranial hypertension patients showed reduced microvascular densities and thinner retinal thicknesses compared with the control group (all *p* < 0.001). Compared with the control group, IH‐P showed reduced microvascular densities and thinner retinal thicknesses (all *p* < 0.001). IH‐P showed reduced SVC density and thinner retinal thicknesses when compared with IH‐WP (*p* = 0.008 for SVC, *p* = 0.025 for RNFL, and *p* = 0.018 for GCIPL). ICP correlated with the microvascular densities and GCIPL thickness in IH patients (*p* = 0.025 for GCIPL, *p* = 0.004 for SVC, and *p* = 0.002 for DVC). A significant association of ICP with SVC (*p* = 0.010) and DVC (*p* = 0.005) densities were also found in IH‐P.

**Conclusions:**

Given the observed differences in these noninvasive retinal imaging markers, further research into their clinical utility in IH is needed.

## Graphical abstract

We showed microvascular attenuations and neurodegeneration correlated with disease duration, reduced visual acuity, and increased intracranial pressure, especially in intracranial hypertension eyes with papilledema. Taken together, our study suggests that OCT/OCTA tool has the potential to detect retinal and microvascular changes in IH. Additionally, OCT/OCTA can be used to study papilledema in IH.

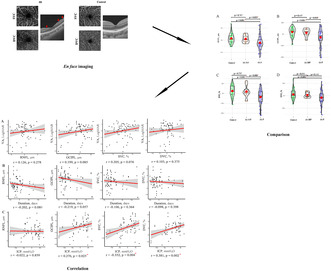

## 1 INTRODUCTION

Intracranial hypertension (IH) is a cerebral disorder that is characterized by manifestations of raised intracranial pressure (ICP); this condition commonly affects obese women of childbearing age. IH patients normally experience headache, pulsatile tinnitus, and diplopia; visual impairment and/or permanent visual loss is the most feared impediment of IH patients and is mainly a result of optic neuropathy that develops in the setting of chronic or severe papilledema.^1^ Clinicians are familiar with the manifestations of the optic nerve head (ONH) such as papilledema, optic disk hemorrhages, and atrophy of the ONH^2^ caused by IH. There is increasing evidence that clinical pointers such as visual impairment and papilledema, strongly suggest that increased ICP affects the ONH, a finding confirmed by numerous research methods: slit lamp, fundus photograph imaging, fluorescein angiography (FA), ultrasonography, and magnetic resonance imaging (MRI).^1^ Nonetheless, retinal manifestations that do occur during the disease are less documented.

Macular exudate, venous stasis retinopathy, and retinal artery occlusion are a few of the retinal manifestations that occur in IH.^3^, ^4^ These retinal changes of IH could lead to a loss in visual acuity (VA) and defects in the visual field, which are distinct from those changes around the ONH caused by papilledema. It is suggested that some of the retinal changes do not respond to the treatments aiming at lowering ICP.^5^ Retinal imaging tools such as optical coherence tomography (OCT) and OCT angiography (OCTA) are suggested to give detailed information on the structural and microvascular changes in the retina. This imaging tool provides a three‐dimensional image of the retinal structure and microvasculature in different layers, which were not visible with previous retinal imaging tools such as fundus photography and FA. OCT/OCTA has been widely applied in neurological diseases such as Alzheimer's disease, ischemic stroke, and multiple sclerosis^6^, ^7^; these studies suggested that the OCT/OCTA tool may have the potential to be used as a screening and monitoring tool to detect retinal changes, which reflect cerebral changes in most neurological diseases. Using the OCT, IH patients were found to have thinner retinal structures when compared to control group^7^, ^8^; in addition, a previous report showed IH patients had reduced microvascular densities around ONH when compared to control group.^9^ These reports suggested the OCT/OCTA tool as a diagnostic and monitoring tool for IH. Thus, adjunctive examination such as OCT/OCTA is critical for the identification of the retinal structural and microvascular changes, and they may help in detecting treatment for lessening or even reversing these retinal changes during the IH process.

It is well documented that papilledema from IH is associated with frequent and extensive ONH damage and retinal impairment^10^, ^11^, ^12^, ^13^ while the pattern of retinal damage in IH eyes without papilledema remains elusive. In addition, whether these retinal changes, particularly the retinal microvasculature, will be affected by the different clinical characteristics such as VA, disease duration, and ICP remain unknown. Therefore, the objective of this study was to report the changes in retinal structure and microvasculature in IH patients when compared to control group, and also in eyes with and without papilledema. Furthermore, we investigated the association between the retinal changes and functional changes in terms of VA, disease duration, and ICP measured via lumbar puncture.

## 2 METHODS

### 2.1 Participants

IH patients were recruited from the Neurology Department of West China Hospital, Sichuan University, China. Patients with IH were diagnosed according to the internationally accepted diagnostic criteria^14^ and had a cerebrospinal fluid pressure greater than 250 mmH_2_O at enrollment as previously reported.^15^ Both IH patients with and without papilledema were enrolled in our study. Exclusion criteria were as follows: (1) History of ischemic stroke, brain tumor, hydrocephalus, and trauma; (2) History of severe mental ailment or psychosis; (3) History of uncontrolled hypertension; (4) Medical ailment requiring concomitant corticosteroids or immunosuppressant therapy. IH included patients with venous sinus stenosis and cerebral venous thrombosis confirmed on digital subtraction angiography (DSA) and magnetic resonance venogram (MRV). Individuals who attended our hospital for an annual checkup without any neurological and ophthalmological disorders were used as control group in our study. Written informed consent was obtained from each participant before enrollment in our study. The research was approved by the Ethics Committee of West China Hospital, Sichuan University, China (No. 2020 [922]). This observational cross‐sectional study followed the tenets of the Declaration of Helsinki.

### 2.2 Lumbar puncture and ICP measurement procedure

The sites of puncture were at L3 ~ 4 intervertebral space, left lateral position, and initial pressure was assessed. Steady readings were recorded when fluctuations in the manometer were stabilized. All lumbar punctures were performed by a skilled neurologist.

All participants underwent ONH and fundus imaging using fundus photography. Photographs of the ONH were evaluated by an ophthalmologist; the diagnosis of papilledema was assessed on ONH images and confirmed by the ophthalmologist.

All participants underwent VA examination using the Snellen chart. Each participant's VA for both eyes was obtained under light and later converted to a logarithm of the minimum angle of resolution (LogMAR).

### 2.3 Swept‐source OCT/OCTA examination

**FIGURE 1:**
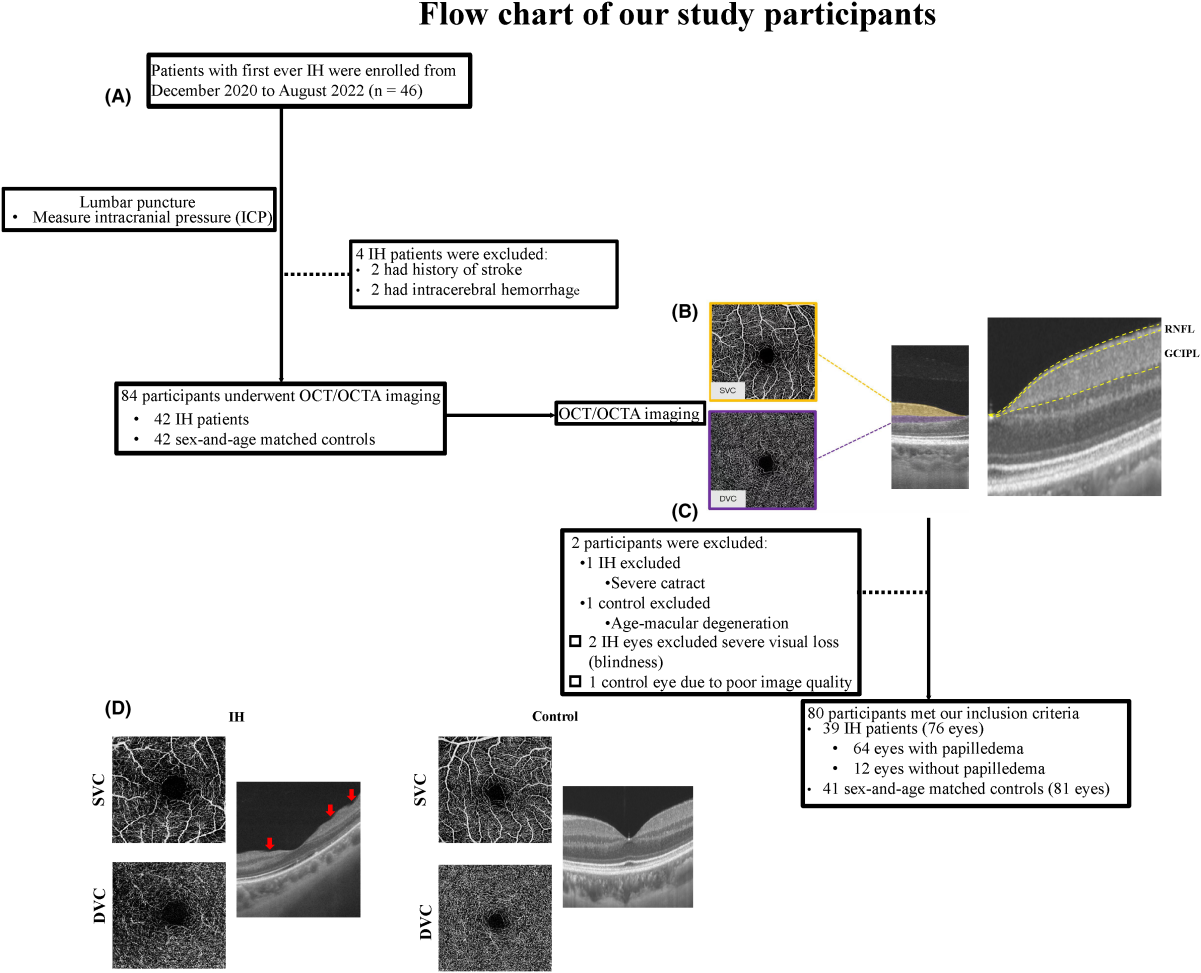
Flow chart, imaging, and segmentation of retina. Figure (A) shows the recruitment, inclusion, and exclusion before retinal imaging. Figure (B) shows structural and microvascular imaging of the retina in a 3 × 3 mm^2^ area around the fovea; the segmentation of the structure and microvasculature of the retina was done by the OCT/OCTA tools. Figure (C) shows the exclusion of participants with ophthalmic diseases after OCT/OCTA examination. Figure (D) shows OCT/OCTA images of an IH patient and a control. Structural imaging of the retina showed IH had retinal folds (red arrows). DVC, deep vascular complex; GCIPL, ganglion cell‐inner plexiform layer; IH, intracranial hypertension; OCT/OCTA, optical coherence tomography/optical coherence tomography angiography examination; RNFL, retinal nerve fiber layer; SVC, superficial vascular complex.

Optical coherence tomography/OCTA (VG200S; SVision Imaging; version 2.1.016) was for retinal imaging. Our previous reports detailed the specifications of the OCT/OCTA tool.^16^, ^17^, ^18^, ^19^ Structural OCT imaging was done with 18 radial B‐scans positioned on the fovea. Each B‐scan was generated by 2048 A‐scans, was 12 mm long, and separated from adjacent lines by 10°. Automatic segmentation of the retinal thickness was done by the OCT tool. Our current study focused on the macular retinal nerve fiber layer (RNFL), and ganglion cell‐inner plexiform layer (GCIPL) in a 3 × 3 mm^2^ area around the fovea as shown in Figure 1. The OCT tool provided the mean thicknesses (measured in μm) of the retinal structure.

The OCTA images covered an area of 3 × 3 mm^2^ around the fovea. The *en face* angiograms of the superficial vascular complex (SVC) and deep vascular complex (DVC) were generated by the OCTA tool. The segmentation of the SVC and DVC slabs was set in the inner two‐thirds and outer one‐third border of GCIPL as shown in Figure 1. Mean percentages (%) of the microvasculature in the SVC and DVC were obtained with an in‐built algorithm in the OCTA tool.

All retinal measurements were done at the macula. OCT/OCTA data displayed in our study followed the OSCAR‐IB quality criteria^20^ and APOSTEL recommendation.^21^

### 2.4 Statistical analysis

The Shapiro–Wilk test was used to examine the normality of our data. Continuous variables were expressed as mean ± standard deviation (SD) for normal data; and median and interquartile ranges (IQR) for skewed data. Categorical variables were presented as frequencies and percentages. To examine the differences between IH patients and control group, the Fisher exact test was used for categorical variables while the t‐test or Kruskal‐Wallis test was used for continuous variables where appropriate. A generalized estimating equation (GEE) was used to compare the OCT/OCTA parameters between IH patients and control group while adjusting for risk factors (age, gender, hypertension, diabetes, dyslipidemia, alcohol, and smoking history). Multiple linear regression was used to describe the correlation between OCT/OCTA parameters and clinical parameters in IH patients (VA, disease duration, and ICP) while adjusting for risk factors. The added variable plot was used to show the partial correlation between clinical insinuations and OCT/OCTA parameters. The *X*‐axis and *Y*‐axis of added variable plots were the residuals of the dependent variable and the independent variable when both of these variables were regressed on the covariates (risk factors). Data analysis and plotting were performed in R version 4.0.3. *p* values less than 0.05 (*p* < 0.05) were considered statistically significant.

## 3 RESULTS

### 3.1 Characteristics of participants

Figure 1 shows the inclusion and exclusion of our study participants. We initially enrolled 46 IH patients and 42 control groups as shown in Figure 1. Our final data analysis included 76 eyes from 39 IH patients (mean age = 34.72 ± 10.18 years) and 81 eyes from 41 control group (37.69 ± 11.46) as shown in Table 1. Out of the 76 eyes from the 39 IH patients, 64 eyes had papilledema (IH‐P) while 12 eyes did not have papilledema (IH‐WP). Table 1 displays the characteristics and clinical information of our participants. IH patients had reduced VA and increased ICP when compared to control group.

**TABLE 1:** Characteristics of study participants.

	IH	Control Group	*p*‐value
Number	39	41	–
Number of eyes	76	85	–
Gender, females	22 (56.4%)	22 (53.66%)	0.982
Age, years	34.72 ± 10.18	37.69 ± 11.46	0.206
Hypertension, *n*	3 (7.69%)	3 (7.32%)	1
Diabetes, *n*	2 (5.13%)	0 (0%)	0.452
Dyslipidemia, *n*	6 (15.4%)	1 (2.44%)	0.098
Smokers, *n*	10 (25.6%)	6 (13.9%)	0.341
Drinkers, *n*	6 (15.4%)	4 (9.76%)	0.672
BMI	25.47 ± 3.11		
VA, LogMAR	−0.511(−1.20 to 0.22)	0 (0.0 to 0.05)	<0.001
Disease duration, days	31 (13 to 42)	–	–
ICP, mmH_2_O	300 (250 to 320)	–	–
Papilledema			
Bilateral, *n*	32 (82.05%)		
Unilateral, *n*	2 (5.13%)		
None, *n*	5 (12.82%)		
Subgroup			
Eyes with papilledema, *n*	64	–	–
Eyes without papilledema, *n*	12	–	–

Abbreviations: BMI, body mass index; ICP, intracranial pressure; IH, intracranial hypertension; LogMAR, logarithm of the minimum angle of resolution; VA, visual acuity.

Table 2 shows the comparison of OCT/OCTA parameters between IH patients and control group.

**TABLE 2:** Comparison of OCT/OCTA parameters between IH patients and control group.

	IH	Control	*p*‐value
RNFL, μm	19.315 ± 2.729	20.791 ± 1.987	<0.001
GCIPL, μm	70.430 ± 14.943	79.362 ± 6.89	<0.001
SVC, %	36.113 ± 8.148	41.304 ± 5.426	<0.001
DVC, %	48.519 ± 5.192	50.691 ± 3.916	<0.001

*Note*: *p*‐values were adjusted for age, gender, vascular risk factors, and intereye dependencies.

Abbreviations: DVC, deep vascular complex; GCIPL, ganglion cell‐inner plexiform layer; IH, intracranial hypertension; RNFL, retinal nerve fiber layer; SVC, superficial vascular complex.

**FIGURE 2:**
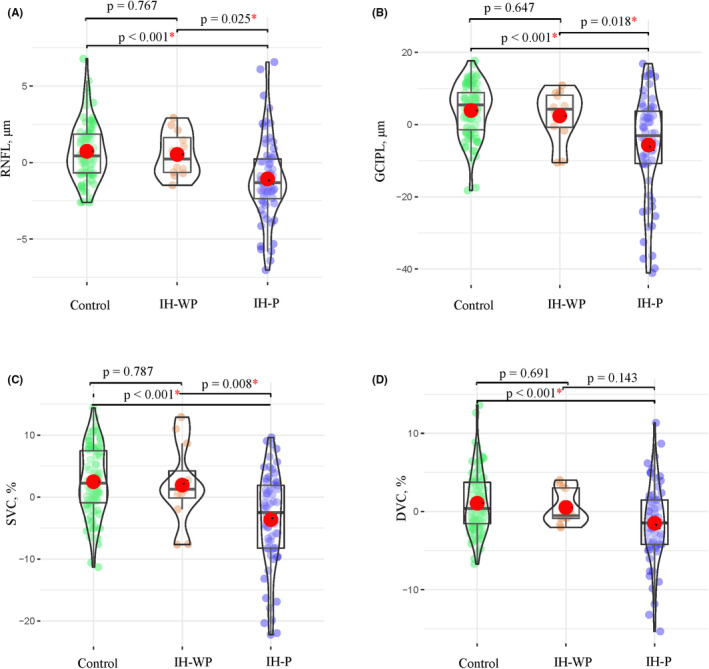
Comparison of OCT/OCTA parameters among IH eyes with papilledema (IH‐P), IH eyes without papilledema (IH‐WP), and control group. Comparison of RNFL (A), GCIPL (B), SVC (C), and DVC (D) among the three groups. The *y*‐axis represents the residuals of the independent variable when regressed on the covariates (risk factors). DVC, deep vascular complex; GCIPL, ganglion cell‐inner plexiform layer; IH, intracranial hypertension; OCT/OCTA, optical coherence tomography/optical coherence tomography angiography examination; RNFL, retinal nerve fiber layer; SVC, superficial vascular complex.

IH patients showed reduced microvascular densities (SVC and DVC) and thinner retinal thicknesses (RNFL and GCIPL) when compared to control group (all *p* < 0.001, Table 2). Compared with control group, IH‐P showed reduced microvascular densities (SVC and DVC) and thinner retinal thicknesses [(RNFL and GCIPL), (all *p* < 0.001, Figure 2)]. IH‐P showed reduced SVC density and thinner retinal thicknesses (RNFL and GCIPL) when compared with IH‐WP (*p* = 0.008 for SVC, *p* = 0.025 for RNFL, and *p* = 0.018 for GCIPL, Figure 2). No significant differences (*p* > 0.05) were found in the OCT/OCTA parameters between control group and IH‐WP.

**FIGURE 3:**
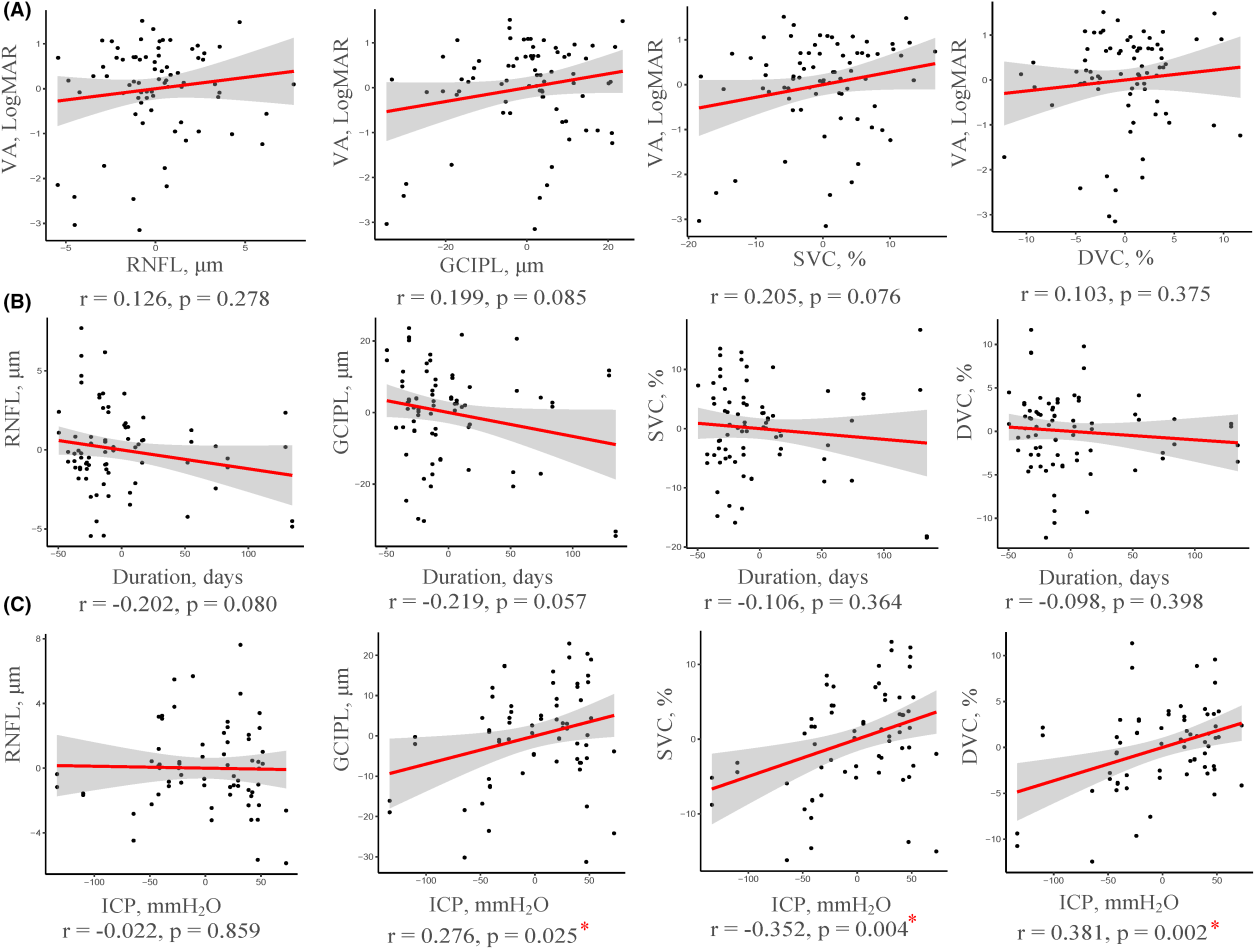
Correlation between OCT/OCTA parameters and clinical parameters in IH patients. Correlation between OCT/OCTA parameters with VA (A), Duration (B), and ICP (C). The X‐axis and Y‐axis of added variable plots were the residuals of the dependent variable and the independent variable when both of these variables were regressed on risk factors. DVC, deep vascular complex; GCIPL, ganglion cell‐inner plexiform layer; ICP, intracranial hypertension; IH, intracranial hypertension; LogMAR, logarithm of the minimum angle of resolution; OCT/OCTA, optical coherence tomography/optical coherence tomography angiography examination; RNFL, retinal nerve fiber layer; SVC, superficial vascular complex; VA, visual acuity.

In IH patients, retinal structures (RNFL and GCIPL thicknesses) correlated (all *p* < 0.001) with microvascular densities (SVC and DVC) as shown in Figure S1. Figure 3 shows the association of retinal parameters measured by OCT/OCTA with the clinical features including VA, disease duration, and ICP in IH patients. ICP was found to correlate with the microvascular densities and GCIPL thickness in IH patients (*p* = 0.025 for GCIPL, *p* = 0.004 for SVC, and *p* = 0.002 for DVC, Figure 3).

In IH‐P, we found ICP correlated with SVC (*p* = 0.010) and DVC (*p* = 0.005) densities (Figure S2). Moreover, SVC density (*p* = 0.039) and GCIPL thickness (*p* = 0.029) correlated with VA while SVC density (*p* = 0.023), RNFL (*p* = 0.019), and GCIPL (*p* = 0.006, Figure S2) thicknesses correlated with disease duration in IH‐P. In IH‐WP, GCIPL thickness (*p* = 0.005, Figure S2) correlated with ICP.

## 4 DISCUSSION

Optic nerve head is the primary ocular site of pathological features in IH. However, retinal changes are reported to be observed during the disease cascade, leading to a loss in VA and defects in the visual field. Using the OCT/OCTA, we aimed to evaluate the structural and microvascular changes in the retina when compared to control group. We showed IH patients had thinner retinal thicknesses and reduced microvascular densities compared with control group. We also showed that increased ICP in IH patients is associated with reduced retinal microvascular densities and thinner GCIPL thickness. Besides, we showed in IH eyes with papilledema, ICP correlated with retinal microvascular densities while VA correlated with retinal thicknesses and SVC density. Importantly, we showed GCIPL thickness correlated with ICP in eyes without papilledema. We suggest that OCT/OCTA might be a cost‐efficient tool to study the subclinical phase in IH patients.

Previous OCT reports showed IH patients have thinner peripapillary retinal nerve fiber layer (pRNFL, structural thickness around the optic nerve head), GCIPL, and thinner macula ganglion cell complex (mGCC) when compared to control group^8^, ^12^, ^22^, ^23^, ^24^; the authors suggested these structural changes are as a result of optic neuropathy. During acute IH, thickening of the pRNFL is associated with the degree of ICP elevation and severity of papilledema.^25^ In our study, IH patients with papilledema had thinner RNFL and GCIPL thicknesses compared with control group, which are in line with previous studies.^24^, ^26^, ^27^, ^28^ RNFL and GCIPL thicknesses are indicators of retinal ganglion cell structure integrity,^29^ where the RNFL comprises retinal ganglion cell axons whereas the GCIPL comprises the cell bodies and dendrites of the retinal ganglion cells.^30^ The ONH is the main ocular site for IH thus, an insult to the ONH causes atrophy ultimately leading to thinning of these neuronal layers during IH^31^ as seen in our study.

We also showed IH patients had significantly reduced SVC and DVC densities compared with control group. The retinal microvasculature is responsible for the metabolic supply of the retinal neurons where thinning of the retinal thicknesses has been observed in IH patients.^29^ Changes in the retinal microvasculature in IH patients may complement the retinal structural indicators. The decreased retinal microvascular densities may be due to the compression of the capillaries by swollen axons at the ONH, thus increasing microvascular resistance. This makes the retinal microvasculature vulnerable to ischemia in IH.

Compared with control group, IH eyes without papilledema showed thinner retinal thicknesses and reduced microvasculature densities though not significant, it still may suggest that subclinical disease activities occur in IH and might propel relapse‐independent disease development. IH eyes with papilledema showed reduced SVC density and thinner retinal thicknesses when compared with eyes without papilledema. This phenomenon may be linked with the pathology of papilledema since the SVC and sub‐retinal thicknesses (RNFL and GCIPL) are susceptible to ONH changes caused by papilledema due to their proximity to the ONH.^29^

A noninvasive, objective measurement of ICP is needed in clinical practice. Vijay et al.^8^ showed a weak correlation between RNFL thickness and ICP measurements (measured by telemetric ICP and lumbar puncture) in IH patients. In the Idiopathic Intracranial Hypertension Treatment Trial (IIH TT), RNFL, total retinal volume, and ONH volume correlated with ICP levels in IH patients.^32^ In our current study, we found that in IH patients, GCIPL thickness, SVC, and DVC densities correlated with ICP measurements. In IH‐WP, GCIPL thickness correlated with ICP while in IH‐P, SVC and DVC densities correlated with ICP measurements. Taken together, we suggest that OCT/OCTA parameters hold promise as a surrogate for ICP.

We found an association between retinal microvascular impairment and neuronal damage in IH eyes with papilledema with worse VA. The integrity of VA has been suggested to affect the quality of life. The major morbidity of IH is visual impairment, which can be progressive and insidious.^33^, ^34^ Loss of vision is often reversible if treatment is introduced in a timely fashion but can be permanent in about 40% of patients.^34^, ^35^ Previous reports^8^, ^24^, ^36^ showed thinning of the mGCC and GCIPL is linked with worse VA in IH patients. Since SVC is responsible for the metabolic supply of the GCIPL, and both play significant roles in vision, our results highlight the importance of preventing early microvascular and neuronal damage and further worsening for protecting vision in IH patients. If confirmed by future reports, this finding may affect clinical decision‐making.

The pathology of IH is characterized by a multifocal mechanism with neurodegeneration and microvascular impairment, which leads to hypoxia and/or ischemia. A noticeable feature of IH is its changeability between patients and heterogeneity in clinical appearances, as well as its tempo of disease progression. We showed SVC density, GCIPL, and RNFL thicknesses in IH eyes with papilledema correlated with the disease duration suggesting that these structures in the retina deteriorate with the duration of the disease.

Taken together, we showed retinal structural and microvascular changes occur in IH patients when compared to control group and these retinal changes were more severe in the eyes of IH patients with papilledema. Importantly, the retinal changes that occur in IH patients were associated with their ICP levels. Recognition of these retinal changes in IH and especially in IH eyes with papilledema is crucial. In our study, we emphasize the importance of involving an ophthalmologist in the patient's care and performing a detailed retinal examination, preferably using OCT/OCTA, which may give detailed information on the retinal changes. Retinal imaging is important and may aid in the diagnosis, monitoring, and treatment of IH patients. The results of our study highlight potential clinical findings that clinicians should be aware of.

We would like to acknowledge some limitations in our study. Our study sample was relatively small, in part because of our strict sampling criteria. Some patients were excluded because of concomitant diseases such as age macular degeneration, macular edema, and severe glaucoma. Another limitation of our study is our retinal parameters was only analyzed globally and not sector‐wise. We did not perform standardized magnetic resonance imaging (MRI) analysis of the brain and optic nerve in our IH patients.

## 5 CONCLUSIONS

In conclusion, we showed subclinical microvascular impairment and neurodegeneration in IH patients compared with control group. In terms of the retinal changes' association with clinical characteristics, microvascular attenuations and neurodegeneration correlated with disease duration, reduced VA, and increased ICP, especially in IH eyes with papilledema. Taken together, our study suggests that OCT/OCTA tool has the potential to detect retinal and microvascular changes in IH. Additionally, OCT/OCTA can be used to study papilledema in IH.

## AUTHOR CONTRIBUTIONS

WRK, LC, JL, and BW designed the study, analyzed, interpreted, and wrote the manuscript. LC, WRK, HW, CY, RP, WT, and JL collected the participants and discussed the results. LC, WRK, and HW involved in retinal imaging. WRK, LC, JL, and BW involved in revision of the manuscript.

## FUNDING INFORMATION

This work was supported by the National Natural Science Foundation of China (82071320, 81901199, 8601022), the 1.3.5 project for disciplines of excellence of West China Hospital, Sichuan University (ZYGD18009), Post Doctor Research Project, West China Hospital, Sichuan University (2021HXBH081), Medical‐Engineering Integration Interdisciplinary Talent Training Fund Project of West China Hospital, Sichuan University and University of Electronic Science and Technology of China (HXDZ22011/ZYGX2022YGRH017).

## CONFLICT OF INTEREST STATEMENT

The authors declare that they have no conflict of interest.

## Supporting information


Figure S1

Figure S2

## Data Availability

The data that support the findings of this study are available upon request from the corresponding author.
