# Supplementary figures and images for: Retinal microvascular and structural changes in intracranial hypertension patients correlate with intracranial pressure
Source: CNS Neurosci Ther. 2023 Jun 8;29(12):4093–101. doi: 10.1111/cns.14298 (PMC10651994; doi:10.1111/cns.14298)

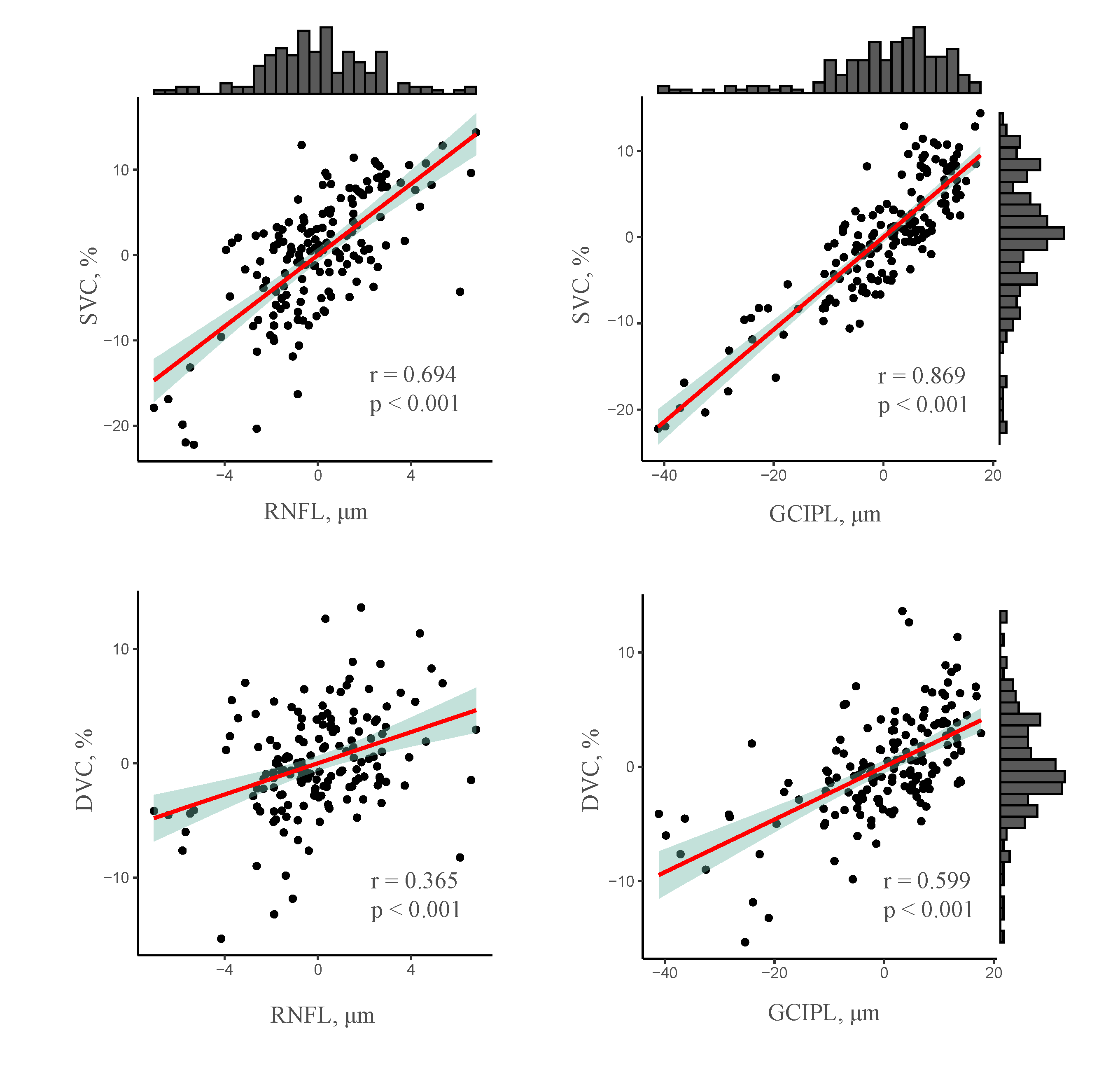

Supplement: Supplementary file 1 — Figure S1 [file CNS-29-4093-s002.tiff]

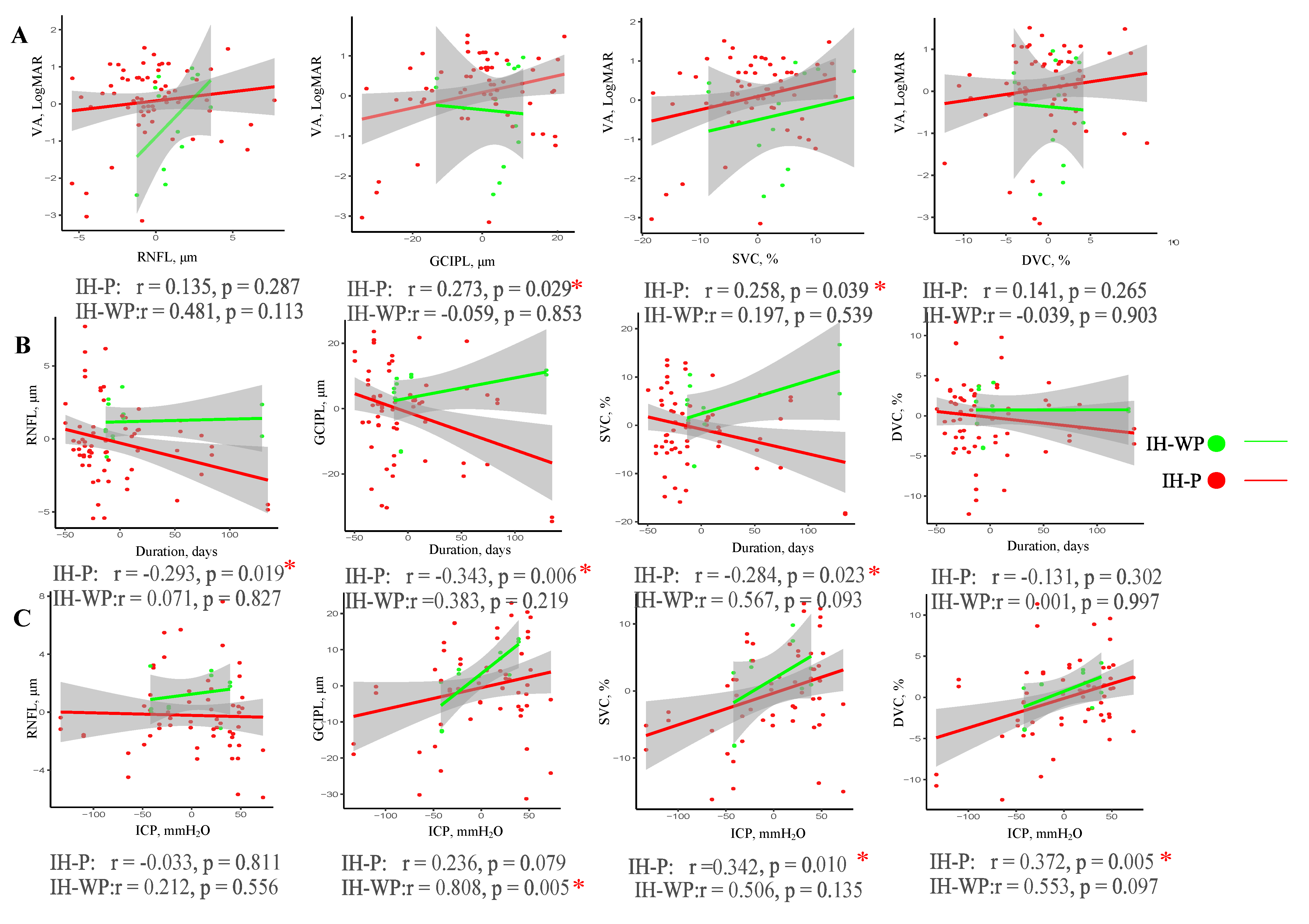

Supplement: Supplementary file 2 — Figure S2 [file CNS-29-4093-s001.tiff]
